# Preoperative Intravenous Desmopressin and Perioperative Blood Loss in Patients Undergoing Major Spine Surgery: Preoperative Intravenous Desmopressin and Perioperative Blood Loss in Spine Surgery Patients

**DOI:** 10.31661/gmj.v14i.3871

**Published:** 2025-10-11

**Authors:** Ali Alizadeh, Mehran Moradi, Mahsa Vafaeimastanabad, Mahsa Hamidiadl

**Affiliations:** ^1^ Qazvin University of Medical Science, Rajaee Hospital, Qazvin, Iran; ^2^ Neurosurgery Departement, Faculty of Medicine, Qazvin University of Medical Sciences, Qazvin, Iran; ^3^ Department of Anesthesiology, Shariati Hospital, Tehran University of Medical Sciences, Tehran, Iran

**Keywords:** Desmopressin, DDAVP, Spine Surgery, Blood Loss, Transfusion, Randomized Controlled Trial

## Abstract

**Background:**

Major spine surgeries often involve significant blood loss, increasing transfusion risks and postoperative complications. Desmopressin, a synthetic vasopressin analog, may reduce bleeding by enhancing clotting factor release, but its efficacy in spine surgery remains understudied. This study evaluated the efficacy of preoperative intravenous desmopressin in reducing perioperative blood loss and transfusion requirements in patients undergoing major spine surgery.

**Materials and Methods:**

A double-blind, randomized, placebo-controlled trial was conducted at Shahid Rajaei Hospital, Iran. Fifty-five patients undergoing major spinal surgery were randomized to receive either 0.3 μg/kg desmopressin (n=25) or placebo (n=30) during anesthesia induction. Primary outcomes included intraoperative blood loss (measured hourly) and transfusion volume. Secondary outcomes included postoperative sodium levels, creatinine, urine output, and hospital stay. Statistical analyses used Student’s t-test, Chi-square, and repeated measures ANOVA.

**Results:**

Desmopressin significantly reduced intraoperative blood loss during the first hour (336.00± 125.43 vs. 398.33±85.58 mL, P=0.034) and second hour (530.95±188.07 vs. 756.67 ± 242.65 mL, P=0.003) compared to placebo. Total transfusion volume was lower in the desmopressin group (724.79 ± 429.06 vs. 1396.55±325.94 mL, P=0.001), with fewer intraoperative packed red blood cell transfusions (16% vs. 40%, P = 0.050). Postoperative creatinine levels were lower with desmopressin (1.16 ± 0.30 vs. 1.40 ± 0.32 mg/dL, P=0.009), but sodium levels and urine output were comparable. Hemodynamic stability and hospital stay did not differ significantly.

**Conclusion:**

Preoperative desmopressin reduced early intraoperative blood loss and transfusion requirements in major spine surgery without significant adverse effects. These findings support its use as a hemostatic adjunct, though further studies are needed to confirm long-term safety and efficacy.

## Introduction

Major lumbar spine surgeries are usually time-consuming and associated with bleeding [1][2][3][4][5][6][7][8][9][10]. Intraoperative bleeding control is of great importance to reduce the need for blood transfusions. In addition, postoperative anemia can increase the risk of myocardial infarction, delay wound healing, and delay patient recovery [11]. Controlling the amount of bleeding and reducing the need for blood transfusions prevents complications of blood transfusion, including infections and hemolytic reactions caused by blood transfusions [8][10]. The need for blood transfusions in spinal surgeries ranges from 40 to 100% [8].

In addition to standard intraoperative techniques, various drugs have been used to achieve hemostasis and reduce bleeding, including DDAVP. This drug is available under the trade name desmopressin and the trade name DDAVP or (D-amino, D-arginine, vasopressin) diamino, diarginine vasopressin, and the trade name Minrin. Desmopressin is a synthetic version of vasopressin, a hormone that reduces urine output, and therefore urine and sodium levels were measured in the current study. Desmopressin was approved for medical use in the United States in 1978 and is listed on the World Health Organization (WHO) List of Essential Medicines as the most effective and safest medicine to be used in a health system and is widely available [12]. It can also be used to treat bleeding from trauma or to prevent bleeding from surgery [12].

It is used to prevent or treat excessive bleeding in patients with bleeding disorders. Desmopressin works by increasing blood clotting, as well as increasing the release of factors VIII and von Willebrand factor from their stores in the body, reducing bleeding [13]. It also reduces urine volume by restricting water intake, so it is considered an antidiuretic. It acts on the collecting duct by binding to the V2 receptor, which signals the translocation of aquaporin channels via cytosolic vesicles to the apical membrane of the collecting duct. The presence of aquaporin channels in the lower nephrons increases the reabsorption of water from the urine, which is passively returned from the nephron and through basement membrane channels to the general circulation. DDAVP also stimulates the release of von Willebrand factor from endothelial cells via the V2 receptor [14][15]. This drug is a vasopressin analog that increases the levels of factor VIII and von Willebrand factor and also causes the release of tissue plasminogen activator [4][11][16][17][18][19]. Given the small number of studies investigating the effectiveness of this drug and also considering that this study has not been conducted in Iran so far, it was conducted with the aim of investigating the effectiveness of DDAVP in patients undergoing major spine surgery. The aim of this study is to compare the effect of intravenous injection of desmopressin before surgery in reducing intraoperative and postoperative bleeding in patients undergoing major spine surgery.

## Materials and Methods

### Study Design

This study was a double-blind, randomized, placebo-controlled clinical trial conducted at Shahid Rajaei Hospital in Qazvin, Iran, involving patients who underwent major spinal surgeries, including degenerative and traumatic cases. Eligible participants were recruited post-admission, and all surgical procedures followed standard discectomy techniques performed by two designated surgeons (Dr. Mehran Moradi and Dr. Sina Abdollahzadeh). Eligible patients were provided written informed consent before being allocated into either the placebo group or the DDAVP (desmopressin) group.

### Participants

Inclusion criteria consist of patients with thoracolumbosacral fractures, degenerative lumbosacral diseases, or traumatic spinal injuries (all age groups). Exclusion criteria include acute cardiac disease, severe heart failure, polydipsia, hyponatremia, and moderate to severe renal failure.

### Randomization and Blinding

Participants were randomized 1:1 to either the DDAVP or placebo group using permuted block randomization with varying block sizes of 4 and 6, generated through the Random Allocation Software (version 2.0). An independent biostatistician prepared sequentially numbered, opaque sealed envelopes containing the allocation assignments to ensure concealment until intervention administration. The study is double-blinded: patients, healthcare providers, surgeons, and data analysts remain unaware of group assignments.

### Interventions

The DDAVP group receives 0.3 µg/kg desmopressin via slow IV infusion over 10 minutes during anesthesia induction. The placebo group receives an equivalent volume of normal saline. The drug’s peak effect occurs at 30 minutes, with a half-life of 4-5 hours.

### Anesthesia Protocol

Preoxygenation is administered 3 minutes before intubation. Induction includes fentanyl (1-2 µg/kg), midazolam (25-50 µg/kg), lidocaine (0.5 mg/kg IV), propofol (2-4 µg/kg), and atracurium (0.5 mg/kg). Maintenance anesthesia consists of propofol (100-150 µg/kg) and remifentanil (0.05 µg/kg). Before incision, bupivacaine (2.5 mg/mL) with epinephrine (1:150,000) is injected subcutaneously. Adjunct medications include dexamethasone (8 mg IV) and ranitidine (50 mg IV).

### Surgical Procedure

All surgeries follow standard discectomy techniques, performed by two designated surgeons. A surgical drain remains for 24 hours postoperatively.

### Outcome Measures

The primary outcome is intraoperative blood loss, measured via gauze weight and hemovac drainage. Secondary outcomes include blood transfusion requirements (calculated using estimated blood volume formulas), postoperative sodium levels, surgery duration, and hospital stay length.

### Sample Size Calculation

Assuming a standard deviation (σ) of 0.5, effect size (d) of 0.8, α=0.05, and 80% power, the required sample size is 24 patients per group (total N=48), calculated using:

n1=n2=2σ2(Z1−α/2+Zβ)2d2≈24n1=n2=d22σ2(Z1−α/2+Zβ)2≈24

### Data Collection and Analysis

Data were analyzed using BM SPSS Statistics for Windows, version 19 (IBM Corp., Armonk, N.Y., USA). Continuous variables (e.g., blood loss, surgery time) were compared via Student’s t-test or non-parametric equivalents; categorical variables (e.g., transfusion need) with Chi-square.

### Ethical Considerations

The study protocol was approved by the institutional ethics committee. All participants provided informed consent, including disclosure of potential risks (e.g., hyponatremia) and the possibility of receiving placebo.

## Results

**Table:** Table1. Baseline Characteristics and Surgical Parameters

**Characteristic**	**Desmopressin (n=25) **	**Placebo (n=30) **	**P-value**
Age (years), M±SD	50.84±10.83	50.33±11.85	0.507
Male gender, n (%)	9 (36.0)	9 (30.0)	0.637
Diabetes mellitus, n (%)	3 (12.0)	10 (33.3)	0.064
Hypertension, n (%)	5 (20.0)	11 (36.7)	0.175
Spinal stenosis, n (%)	15 (60.0)	26 (86.7)	0.024*
Operative time (min), M±SD	135.86±36.45	135.33±36.55	0.958
Surgical levels, M±SD	3.56±0.87	3.63±1.67	0.844

**Table:** Table2. Blood Loss and Transfusion Outcomes

**Outcome**	**Desmopressin (n=25) **	**Placebo (n=30) **	**P-value**
**Blood loss (ml), M±SD **			
- First hour	336.00±125.43	398.33±85.58	0.034*
- Second hour	530.95±188.07	756.67±242.65	0.003**
- Third hour	908.33±380.02	1050.00±70.71	0.636
**Transfusion**			
- Total volume (ml), M±SD	724.79±429.06	1396.55±325.94	0.001**
- Intraoperative PRBC, n (%)	4 (16.0)	12 (40.0)	0.050*
- Postoperative PRBC, n (%)	2 (8.0)	3 (10.0)	0.998

**Table:** Table3. Laboratory Parameters

**Parameter**	**Desmopressin (n=25) **	**Placebo (n=30) **	**P-value**
**Sodium (mEq/L), M±SD **			
- Preoperative	138.28±2.55	137.83±3.96	0.06
- Postoperative	139.32±3.68	138.40±4.56	0.442
**Creatinine (mg/dL), M±SD **			
- Preoperative	0.90±0.19	0.95±0.24	0.442
- Postoperative	1.16±0.3	1.40±0.32	0.009**
**Urine output (ml), M±SD **			
- Intraoperative	196.60±96.7	166.66±46.41	0.126
- 24-hour postoperative	982.00±816.28	741.50±154.58	0.119

**Table:** Table4. Hemodynamic and Clinical Outcomes

**Timepoint**	**Desmopressin (n=25) **	**Placebo (n=30) **	**P-value**
**SBP (mmHg), M±SD **			
Preoperative	136.00±7.03	136.66±9.45	0.578
6h post-transfer	114.48±8.08	110.56±5.91	0.041*
**Hospital stay (days), M±SD **	1.82±0.39	2.29±2.21	0.392

The study randomized 55 patients undergoing spinal surgery to receive either desmopressin (n=25) or placebo (n=30). Baseline characteristics were well-balanced between groups. The mean age was 50.84 years (SD = 10.83) in the desmopressin group compared to 50.33 years (SD=11.85) in the placebo group (P=0.507). Gender distribution showed 36% males in the desmopressin group versus 30% in controls (P=0.637). Comorbidity profiles were similar, with no significant differences in diabetes mellitus (12% vs 33%, P=0.064) or hypertension (20% vs 36.7%, P=0.175), though spinal stenosis was more prevalent in controls (60% vs 86.7%, P=0.024). Surgical parameters including operative duration (135.86 ± 36.45 vs 135.33 ± 36.55 minutes, P=0.958), number of surgical levels (3.56±0.87 vs 3.63±1.67, P=0.844), and number of screws placed (7.04±1.74 vs 7.27±3.34, P=0.761) showed no significant between-group differences (Table-1).

### Blood Loss and Transfusion Requirements

Intraoperative blood loss was significantly reduced in the desmopressin group during the first hour (336.00±125.43 ml vs 398.33±85.58 ml, P=0.034) and second hour (530.95 ± 188.07 ml vs 756.67 ± 242.65 ml, P = 0.003) of surgery. Total blood transfusion volume was markedly lower with desmopressin (724.79 ± 429.06 ml vs 1396.55 ±325.94 ml, P=0.001). The need for intraoperative packed red blood cell transfusion occurred in 16% of desmopressin patients compared to 40% of controls (P=0.050), while postoperative transfusion rates were similar between groups (8% vs 10%, P=0.998) (Table-2).

### Laboratory Parameters and Renal Function

Serum sodium levels remained stable in both groups throughout the perioperative period (preoperative: 138.28 ± 2.55 vs 137.83 ± 3.96 mEq/L, P=0.060; postoperative: 139.32 ± 3.68 vs 138.40 ± 4.56 mEq/L, P=0.442). While baseline creatinine levels were comparable (0.90 ± 0.19 vs 0.95 ± 0.24 mg/dL, P=0.442), postoperative values were significantly lower in the desmopressin group (1.16 ± 0.30 vs 1.40 ± 0.32 mg/dL, P =0.009). Urine output showed no significant differences either intraoperatively (196.60 ± 96.70 vs 166.66 ± 46.41 ml, P=0.126) or during the first 24 postoperative hours (982.00 ± 816.28 vs 741.50 ± 154.58 ml, P=0.119) (Table-3).

### Hemodynamic Stability and Clinical Outcomes

Repeated measures ANOVA of systolic blood pressure measurements revealed no significant between-group differences in hemodynamic patterns (P=0.535). The only significant blood pressure difference occurred at 6 hours post-transfer to the ward (114.48 ± 8.08 vs 110.56 ± 5.91 mmHg, P=0.041). Hospital length of stay was comparable between groups (1.82 ± 0.39 vs 2.29 ± 2.21 days, P=0.392) ((Table-4).

## Discussion

This study aimed to investigate the effectiveness of DDAVP in patients referred to Shahid Rajaee Hospital in Qazvin in 2018-2019 undergoing major spinal surgery.

In a study conducted in 2015 by Lie Jin et al., factors such as age, gender, and time of surgery were compared between the two placebo and patient groups. There was no significant difference between the two groups. In our study, there was no difference between the placebo and patient groups in terms of these characteristics, according to the statistical data. Also, in the mentioned study, the amount of bleeding was measured and analyzed 6 and 24 hours after surgery. In the first 6 hours in the desmopressin group, the amount of bleeding was significantly reduced (P=0.023) and there was no difference between the two groups 24 hours after surgery. However, in our study, which has addressed this issue in more detail, the amount of bleeding was measured during the first hour, second hour, third hour, during the operation, and up to 3 hours after the operation. According to the results obtained, the amount of bleeding during the first (P=0.034) and second (P=0.003) hours during the operation in the desmopressin group was much less than the amount of bleeding in the placebo group, but There was no statistically significant difference in the third hour during the operation and up to 3 hours after the operation. In addition, the factors examined by Lie Jin et al. included hemoglobin level, urine output during and after the operation, which did not show any difference between the two groups mentioned in their studies. In our study, although these factors, for example, hemoglobin level was measured 6 hours before the operation and 6, 12, and 24 hours after the operation, no significant difference was seen between the hemoglobin volume between the two desmopressin and placebo groups, which were the same in this respect [20].

In the study conducted in 1998 by LETTS et al., it was proven that factors such as age and gender are not involved in the effectiveness of desmopressin, as was the information obtained from our analysis. But in this study, other factors such as bleeding time, hemoglobin, factor 8, prothrombin, etc. were measured. The reduction in the average bleeding time before and after desmopressin injection indicates the effect of this drug. However, in our study, the amount of bleeding was examined and the bleeding time was not our concern. However, the results regarding hemoglobin differ from our study. In our data, the amount of hemoglobin 6 hours before the operation and 6, 12, and 24 hours after the operation did not differ significantly in the two groups. However, in the study by LETTE et al., the amount of hemoglobin in the desmopressin group decreased significantly, and the examination of the amount of bleeding also showed a decrease in the amount of bleeding in both studies [21][22][23].

In a study conducted in 1995 by G. Patrick et al., factors such as age, sex, diabetes, and creatinine were examined. Similar to the data obtained from our study, it was found that none of these factors had any effect on the effectiveness of desmopressin in reducing bleeding volume. However, the blood pressure in both studies showed different results and was not statistically different between the groups. The study of blood pressure changes using repeated measure ANOVA test indicated that there was no difference in the change in bleeding volume during the first three hours after surgery (P=0.535), whereas in the study by G. Patrick et al., blood pressure did not differ at different stages. Another factor that differs in these two studies is the amount of bleeding, which decreased in our study, but in the study by G. Patrick et al., no difference in bleeding was observed between the two desmopressin and placebo groups [22][24][25][26][27][28][29].

In a study conducted in 1997 by MARY C et al., factors such as age, degree of scoliosis, hemoglobin before and after surgery, duration of surgery, platelet count, and bleeding volume, etc. were measured. In the end, it was determined that none of these factors had any effect on the effectiveness of desmopressin. There was no significant difference between the two groups of desmopressin injection and the placebo group. However, in our study, apart from the amount of bleeding, none of the factors had any effect on the effectiveness of desmopressin [24][25].

A study conducted in 1992 by Guay j et al., like our study, factors such as gender, age, duration of surgery, and urine output 1, 2, 3, 4, and 24 hours after surgery were measured. Like our study, there was no significant difference between the two groups. However, bleeding time was measured in this study, and no difference was observed between the two groups, although we did not examine this factor [21].

In this study, analysis of primary and background information between the two desmopressin and placebo groups, including age, gender, duration of surgery, prevalence of hypertension, prevalence of spinal stenosis, average number of surgical levels, average number of screws inserted during surgery, and number of laminectomy levels, showed that there was no significant difference between the two groups, except for the average prevalence of diabetes mellitus.

In the comparison of intraoperative and postoperative hemodynamic indices between the two placebo and drug groups, including intraoperative bleeding volume during the first, second, and third hours of surgery, volume of whole blood infused during surgery, need for packed cell infusion during and after surgery, blood sodium concentration 12 hours before and after surgery, intraoperative urine output and 24 hours after surgery, BUN during 12 hours before and after surgery, serum creatinine during 12 hours after surgery, length of hospitalization from surgery to discharge, mean serum hemoglobin 6 hours before and after surgery and 12 and 24 hours after surgery, it was shown that the amount of bleeding in the first and second hours of surgery in the group that received desmopressin was lower than in the placebo group, which was consistent with a study by Kobrinsky NL et al. in 1987 and studies conducted in 2015 by Mr. Lie Jin et al. and a study by LETTS M et al. in 1998 aimed at estimating the effect of DDAVP on bleeding [20][23][30]. However, during the third hour, the two groups did not differ significantly, which is consistent with a study conducted by Guay j et al. in 1992 to evaluate the effect of DDAVP on reducing bleeding in patients undergoing spinal surgery with idiopathic scoliosis, in terms of the lack of effect of desmopressin on the amount of bleeding [21]. The desmopressin group required a lower volume of whole blood infused intraoperatively and a smaller amount of packed cell injection intraoperatively, but there was no significant difference in the need for packed cell injection postoperatively. The mean blood sodium concentration 12 hours before surgery in patients in the desmopressin and placebo groups was statistically significantly higher in the desmopressin group. However, the mean blood sodium concentration 12 hours after surgery, the mean urinary output during surgery and 24 hours after surgery, the mean BUN during 12 hours after surgery, and the mean serum creatinine during 12 hours before and after surgery were not statistically different between the two groups. However, the mean serum creatinine within 12 hours after surgery was significantly higher in the control group. The mean length of stay from surgery to discharge, serum hemoglobin 6, 12, and 24 hours after surgery were not statistically different between the two groups, which is consistent with a study conducted in 1995 by Mr. G. Patrick Clagett [29].

## Conclusion

In the present study, the reduction in bleeding during the first and second hours of surgery in the group that received desmopressin was greater than placebo. Also, the mean volume of whole blood infused and the frequency of packed cell injections during surgery were reduced in the group that received desmopressin. The blood sodium level was higher in the group that received desmopressin 6 hours and 12 hours before surgery. The blood pressure was also lower in the group that received desmopressin up to 6 hours after transfer to the ward.

## Conflict of Interest

None.
